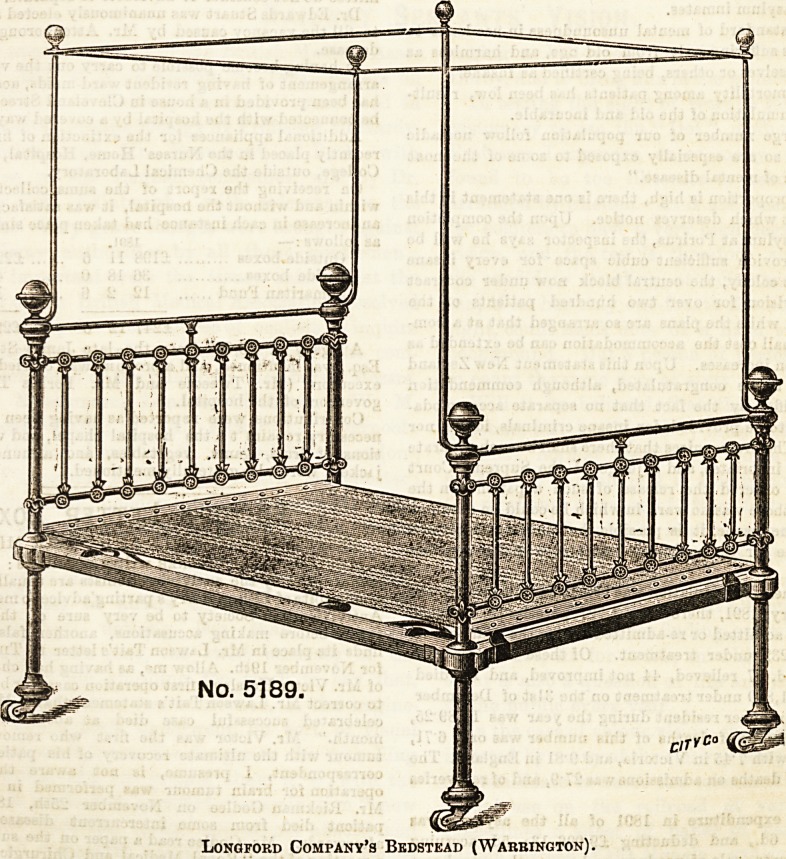# Bedsteads I

**Published:** 1892-12-03

**Authors:** 


### PRACTICAL DEPARTMENTS.

### BEDSTEADS.—I.

When we begin to study the subject of bedsteads suited for institution use, we are surprised to find what a number of varieties already exist, and yet new patterns are constantly brought forward, and some of these are really excellent. The heavy, clumsy ironwork, apparently designed for the purpose of providing substantial resting places for dust, has given place to a simpler and lighter style. The iron laths have been superseded by spring bottoms, and the bar underneath is but seldom seen, and both these changes are greatly to the advantage of the bed-makers. A few years ago flock beds were very general, and they tried the nurses as much as they did the patients, only in different ways, the unevenness and lumps affecting the latter, and the general untidy appearance distressing the former; and certainly the "springs" of modern times are a vast improvement on any previous fashions, for they are very comfortable, easy to keep clean, and quickly put together or taken down.

Some of the bedsteads shown by Atkinson, Westminster Bridge Road, are very strong and simple in construction, consisting of only three pieces, the head piece and a low foot piece, with a bottom of galvanised springs, mesh attached. No dust-accumulating iron bar beneath, and everything firm in make and easily fixed.

There are some institutions where a foot rail may not be considered necessary, but it is absolutely indispensable for sick people. It can be quite a low one if preferred, but it ought always to exist. When persons are weak and ill they have always a tendency to slip down in the bed, in spite of pillows and other supports, and therefore it should not be possible for the bedding to slide also, as this considerably aggravates the patient's discomfort and the nurse's difficulties. The advantage of a foot piece to a bed is by no means to be measured by its size, for a small one may be a very large comfort. These, like the bedsteads, vary in shape and style.

Of course, it is well to avoid all sharp corners or points that can be productive of danger to restless persons or to straying drapery. Bow ends are therefore good, and round bars are also advantageous for these reasons. The castors to bedsteads should be very carefully considered. Many that look sufficiently strong when viewed on an empty bedstead, prove very insufficient for the additional weight of a substantial mattress and bed clothes, as well as a helpless patient of average size.

brass knobs, render the bedstead a peculiarly suitable one for sick wards. It provides facilities for a steam tent, which is frequently required for medical cases of illness, in a manner specially satisfactory to practical nurses. Of course two or three sets of poles would form an ample supply for one ward, care being taken that they fit every bedstead therein. Another pattern by the same makers is known as "The Salford Design," having been supplied to the Royal Infirmary, Salford. Here again we see an admirable wire mattress, and the 1½-inch tubing is of the best quality, whilst the joints of malleable iron render the framework practically indestructible. The pulley or elevator appears strong and well suited for the patient's convenience.

*(To be continued.)*

Longford Company's Bedstead (Warrington).

Therefore the castors should be thoroughly good and of a size to permit of the bed being easily moved without jar or disturbance to the occupant.

A sheet-iron head-piece is sometimes chosen on account of its being thought safer for certain kinds of patients, and it has some advantages over bars, but it is very much heavier looking, and has no special advantages to recommend it for most institutions or general hospitals.

The bedstead shown here is made by the Longford Company at Warrington, and is recommended for use in hot climates, as the rods and poles are specially designed for the support of mosquito curtains.

However, the strong, plain ironwork, and the simplicity of the method which fixes the rods securely beneath the

## Figures and Tables

**Figure f1:**